# Lung Organoids and Their Use To Study Cell-Cell Interaction

**DOI:** 10.1007/s40139-017-0137-7

**Published:** 2017-04-24

**Authors:** Marko Z. Nikolić, Emma L. Rawlins

**Affiliations:** Gurdon Institute, University of Cambridge, Tennis Court Road, Cambridge, CB2 1QN UK

**Keywords:** Organoids, Lung progenitors, Mouse lung, Human lung, iPSCs

## Abstract

**Purpose of Review:**

The lung research field has pioneered the use of organoids for the study of cell-cell interactions.

**Recent Findings:**

The use of organoids for airway basal cells is routine. However, the development of organoids for the other regions of the lung is still in its infancy. Such cultures usually rely on cell-cell interactions between the stem cells and a putative niche cell for their growth and differentiation.

**Summary:**

The use of co-culture organoid systems has facilitated the in vitro cultivation of previously inaccessible stem cell populations, providing a novel method for dissecting the molecular requirements of these cell-cell interactions. Future technology development will allow the growth of epithelial-only organoids in more defined media and also the introduction of specific non-epithelial cells for the study of cell interactions. These developments will require an improved understanding of the epithelial and non-epithelial cell types present in the lung and their lineage relationships.

## Introduction

Organoids are defined as three-dimensional (3D) structures derived from stem cells and consist of organ-specific cell types which self-organise through cell sorting and spatially restricted lineage commitment in a manner reminiscent of the native organ with some degree of organ functionality [1, 2]. Organoids have also been referred to as “mini-organs” and enable in vitro modelling of organ development, disease modelling and drug screening. The 3D culture preserves native DNA integrity and prevents the cells from being transformed [13]. Organoids were first successfully derived from mouse small intestine using single Lgr5^+^ stem cells [3]. These organoids were entirely epithelial illustrating that organoids can be built without a non-epithelial cellular niche. The organoid was structured as crypt-villus units, with a similar stem cell hierarchy to in vivo, showing epithelial cell interactions are sufficient for the creation of crypt-villus units. Further work on cell-cell interactions using these intestinal organoids showed that essential niche signals are provided by Paneth cells, which are found interspersed between Lgr5^+^ stem cells [4].

Organoid growth requires the initiating stem cell population to self-renew, to increase organoid size, and to differentiate. Organoids have been successfully cultured from multiple endoderm-derived organs including the adult mouse stomach [5], mouse colon [6], human colon [7], mouse pancreas [8], mouse liver [9], mouse prostate [10], human prostate [10], human intestine [11], and mouse embryonic pancreas [12]. In most of these studies, the same tissue culture medium supported both stem cell self-renewal and differentiation, for example intestinal stem cells self-organise efficiently into organoids and differentiate [3, 4]. By contrast, the adult liver and pancreas organoids can be expanded but do not differentiate easily yet [13, 14]. Similarly, mouse embryonic pancreas progenitors were expanded in a self-renewing medium and then switched to a differentiation medium for maturation [12, 15, 16]. This switch in medium composition may be particularly important for organoids derived from embryonic progenitors as such cells are typically reliant on extrinsic signalling from the adjacent mesenchyme in vivo [17, 18]. Cell-cell interactions within organoid cultures are likely to be just as important for differentiation as they are in vivo, through a process termed the “community effect”. This is now a well-established phenomenon in which the differentiation ability of a cell is enhanced by neighbouring cells differentiating in the same way simultaneously [19].

Lung organoids have been successfully grown from the embryonic lungs [20–24], mouse adult lungs [25, 26, 27••, 28], human adult lungs [25, 28], and human iPSC (induced pluripotent stem cell) derived lung progenitors [29, 30, 31••]. This review will first introduce the lung as an organ with its various cell types and then evaluate the literature involving 3D cultures derived from lung stem cells, which we are referring to as organoids if they have demonstrated self-renewal capacity, or spheroids if self-renewal has not yet been achieved. We focus particularly on their currently widespread use for studying cell-cell interactions.

## The Lung and Its Cell Types

Lung diseases account for the third highest mortality of non-infectious disease deaths and lung cancer is the most common cancer worldwide [32]. The mortality is partly due to irreversible destruction of lung tissue and the associated inability to meet the demands for lung transplantation. In order to address this, characterising the growth requirements of the different stem cell populations in both the adult and developing lungs is essential. One approach is by studying cell-cell interactions in organoids.

The lung is a complex structure of branched epithelial-lined airways and endothelial-lined blood vessels which unite at the alveoli for gas exchange. The lung is surrounded by pleura; referred to as the mesothelium in mouse [33, 34]. The trachea divides at the carina forming the left and right main stem bronchi. Each of which divides further into secondary, or lobar, bronchi and subsequently into progressively smaller bronchi and bronchioles, until the smallest bronchioles connect to the alveoli [35].

**Fig. 1:**
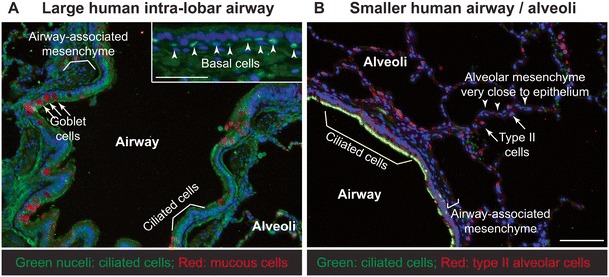
Cellular organisation of the human lung. The cellular complexity of the human lung epithelium. **a** Section through large human intra-lobar *airway* stained to show ciliated cell nuclei (FOXJ1^+^; *green*) and mucous-producing *Goblet cells* (SCGB1A1^+^; *red*). It should be noted that *Goblet cells* are less prominent than *Ciliated cells*. The third major airway epithelial cell type (*Basal cells*) is shown in *green* in the *inset* as TRP63^+^ cells (*arrowheads*). The green background (due to auto-fluorescence from the tissue) nicely illustrates the airway-associated mesenchyme which has yet to be extensively characterised. **b** Section through a smaller human *airway* also showing the adjacent alveolar region. Here, cilia are visualised using an acetylated-tubulin antibody (*green*). *Goblet cells* are not shown, but are found at similar frequency to **a**. Note that the *airway-associated mesenchyme* is less extensive than in that in **a**. *Type II* alveolar cells are visualised using pro-SFTPC staining (*red*). The mesenchyme in the alveolar region is even less extensive and tightly associated with the epithelium. *Scale bars*: **a** 100 μm; **b** 50 μm (*inset*)

The human tracheobronchial airway is mostly lined by pseudostratified epithelium in which each cell type makes contact with the basement membrane. Below the basement membrane, blood vessels, smooth muscle, cartilage, extracellular matrix-producing fibroblasts, and nerves are found. The height of luminal epithelial cells and the proportion and density of the different cell types vary along the proximal-distal axis and between human and mouse [33]. The major airway epithelial cell types are basal, secretory (primarily goblet in human and club in mice), and ciliated cells, although neuroendocrine and brush cells form a more minor component (Fig. 1). Basal cells are present throughout human conducting airways but are confined to the trachea and primary bronchi of mice [36, 37].

The alveolar epithelium consists of type I and type II alveolar cells (AC1 and AC2) which are surrounded by capillaries and multiple different types of fibroblasts [38–40]. AC1 cells are flat, highly extended, and specialised for gas exchange as they cover more than 95% of the gas exchange surface area; whereas, AC2 cells are cuboidal, are more common, and are specialised for surfactant protein production [33, 38, 40, 41] (Fig. 1). Blood vessels are lined with vascular endothelial cells and also contain pericytes and vascular smooth muscle depending on size. The lymphatic system of the lung is poorly characterised and consists of lymphatic endothelial cells and associated stroma. The cellular interactions between alveolar macrophages, lung dendritic cells, and epithelial cells are thought to be crucial in restraining immune damage after infection or any other kind of damage to the epithelial barrier [42]. Controversy exists about the function of myofibroblasts and lipofibroblasts and other putative mesenchymal fibroblast populations. Their markers have not yet been defined, but they are thought to play important roles in lung alveolar development and maintenance [43].

The lung is a complex organ with numerous distinct cell types. How these cells interact in development, homeostasis, and disease is a key research question and organoids provide a platform for investigating some of these cell-cell interactions (Table 1).

**Table 1:** Lung organoids and their use to study cell-cell interactions

Type	Organoid-forming cell	Supporting cell types	Organoid cell type composition	Cell-cell interactions
Lung organoids from embryonic lungs				
Human embryonic [24]	Human embryonic lung fibroblasts	Human umbilical vein endothelium (HUVECs) and small airway epithelial cells	Fibroblasts, endothelium, airway epithelium	Mesenchyme-epithelium-vascular endothelium
Lung organoids from iPSC-derived lung progenitors				
Mouse iPSC [63]	Endoderm-induced embryoid bodies	Rat or mouse embryonic lung mesenchyme	Alveolar fated epithelium; fibroblasts	Epithelial-mesenchymal
Human iPSC [29]	CMP+/NKX2.1+ VAFE lung progenitors	Human foetal lung fibroblasts (17.5-week gestation, exact cell population unknown)	Alveolar fated epithelium; fibroblasts	Epithelial-mesenchymal
Human iPSC [31••]	VAFE lung progenitors	Unknown mesenchymal cell type(s) derived from the iPSCs	Bronchiolar (basal, club, ciliated); alveolar progenitors; SMA+ and SMA− mesenchyme	Epithelial-mesenchymal
Human iPSC cultured in vivo on PLG scaffold [44]	VAFE lung progenitors	Unknown mesenchymal cell type(s) derived from the iPSCs	Bronchiolar (basal, club, ciliated, mucous); cartilage, myofibroblasts, smooth muscle; vasculature	Epithelial-mesenchymal-endothelial
Lung organoids from adult mouse lungs				
Mouse adult tracheal [73]	Adult tracheal basal cells	Adult tracheal club cells	N/A—organoid-forming abilty of each cell type assessed	Epithelial-epithelial
Mouse adult alveolar [28]	Adult AC2 cells	PDGFRA+ alveolar fibroblasts	AC2 and AC1; fibroblasts	Epithelial-mesenchymal
Mouse adult alveolar and bronchiolar [27••]	Adult putative BASC stem cells	Expanded mouse lung endothelium	Bronchiolar; or alveolar; or bronchoalveolar organoids	Epithelial-endothelial
Mouse adult small airway [77]	Adult small airway club cell populations	Adult mouse lung stromal (non-epithelial) primary cells	N/A—spheroid-forming abilty of each cell type assessed	Epithelial-mesenchymal
Mouse adult small airway [45]	Total adult lung epithelial cells	Adult mouse lung expanded fibroblasts (passage 1), or primary adult lung endothelial cells	Bronchiolar; or alveolar; or bronchoalveolar organoids—depending on identity of starting stem cells	Epithelial-mesenchymal and epithelial-endothelial
Mouse adult rare alveolar progenitors (KRT5/p63+) [46]	Rare alveolar progenitors	Adult mouse lung stromal (non-epithelial) primary cells	N/A—spheroid forming abilty assessed	Epithelial-mesenchymal

## Organoids Derived from Embryonic Lungs

In the mouse, lung development starts after establishment of the primary germ layers with the two primary endoderm buds becoming visible at around E9.5 [33]. Molecularly, the respiratory lineage can be first identified by expression of the transcription factor Nkx2.1 in the ventral anterior foregut endoderm [34, 47] as early as E8.25 [48]. The mouse lung then develops, similar to a human’s, as a blind-ended tube which branches multiple times, a process governed by a complex network of transcription factors and signalling pathways. The important stem cell, or progenitor, population in the developing embryonic mouse lung is found in the distal branching tip [49]. These epithelial tip cells comprise a multipotent progenitor population giving rise to both bronchiolar and alveolar cells [49–51].

Whole E12.5 mouse lungs can be cultured in vitro on an air-liquid interface in 2D where they will both grow and differentiate [52–54]. These explant cultures could be described as the “ultimate mini-organs” as they contain all of the cells needed for normal lung development. However, their size, cellular complexity, and growth in a 2D plane, rather than in a 3D, makes them unsuitable for many experiments.

Mixed cell populations obtained from dissociated whole E17.5 mouse lungs have been grown in 3D on various matrices [20–22]. Such cultures self-organise into spheroids containing branched epithelial structures surrounded by mesenchyme grow and show signs of alveolar and bronchiolar differentiation. However, self-renewal has not been tested and detailed cellular phenotyping has not been performed. These cultures have been used to test the effects of growth factors, such as FGFs and VEGF-A [20, 21], or for direct differentiation to alveolar structures [22]. Since whole foetal lungs were minced and then grown as spheroids, the culture is limited by the lack of a defined starting population. However, these spheroids could be useful as a tool for studying morphogenesis in vitro.

A recent advance is the formation of mesenchymal organoids consisting of human foetal lung (18- to 20-week gestation) fibroblasts grown on collagen-coated alginate beads in 3D units [23]. These were exposed to varying oxygen concentrations to mimic the oxygen tension experienced by premature neonates for disease modelling. Proof of concept experiments suggest that this system has the potential to incorporate additional lung cell types, including airway epithelial and endothelial cells [24]. It will be interesting to see if this bioengineering approach with defined cell types will be able to accurately recapitulate cell interactions and potentially even disease phenotypes.

## Organoids from iPSC-Derived Lung Epithelial Cells

iPSCs are derived through reprogramming adult somatic cells by introducing pluripotency transcription factors [55]. The differentiation of patient-specific iPSCs into any relevant cell type can provide a platform for disease modelling, drug screening, and cell-based therapies. Several groups have successfully differentiated iPSCs into lung progenitors [56, 57] and more differentiated alveolar and bronchiolar lung epithelial cells [58–63]. All of these protocols used directed differentiation of iPSCs as monolayers in 2D culture systems and attempted to recapitulate normal development as described in the mouse lung developmental literature. Briefly, this involves differentiation into definitive endoderm, followed by anterior and ventral foregut endoderm. Ventral anterior foregut endoderm (VAFE) Nkx2.1^+^ lung progenitors are subsequently differentiated into bronchiolar or alveolar cell fates using a stage-specific combination of growth factors. Recent efforts have been made to increase the maturity of iPS-derived differentiated cells by 3D differentiation as organoids [31••, 64]. This approach has been successful to some extent for both alveolar [29] and bronchiolar differentiation, where there was evidence of effective differentiation into ciliated cells with beating cilia [30].

The first published attempt at deriving alveolar organoids from iPSC-produced human lung progenitors used carboxypeptidase M (CPM) as a cell-surface marker to specifically isolate VAFE iPSC-derived lung progenitors. These were then seeded into Matrigel with human foetal lung fibroblasts obtained at 17.5 weeks of gestation and an alveolar-specific combination of growth factors [29]. These NKX2.1^+^ organoids contained small numbers of Aquaporin5^+^ AC1 cells and pro-SFTPC^+^ (SPC) AC2 cells, the latter of which contained some structures reminiscent of lamellar bodies when analysed by transmission electron microscopy. Interestingly, CPM^+^ iPSC-derived lung progenitors cultured without human foetal lung fibroblasts were unable to produce SPC^+^ cells, suggesting that the fibroblasts produce essential signal(s) for alveolar identity. Although the AC2-like cells were produced at low efficiency and are likely to be very immature, this co-culture is an encouraging strategy which could be improved upon by improved characterisation of the fibroblast cell type(s) and optimised culture conditions.

The same group used a similar strategy of isolating CPM^+^ iPSC-derived lung progenitors followed by organoid culture to produce bronchiolar lung organoids. In this case, there was evidence of differentiation into ciliated cells with beating cilia, as well as neuroendocrine, secretory, and basal cells [30]. Interestingly, there was no ciliated cell differentiation in 2D culture, suggesting that 3D organoid culture enabled successful differentiation into functional ciliated cells. However, ciliary beating was not fully synchronised as required for unidirectional flow and mucociliary clearance. This suggests functional immaturity, similar to the previous studies, or the lack of a directional cue. Organoids consisted only of epithelial cells without any evidence for mesenchymal differentiation.

Another study differentiated human pluripotent cells into VAFE spheroids [31••]. These foregut spheroids were then differentiated into lung organoids with both bronchiolar and alveolar regions and maintained in culture for over 3 months. Bronchiolar structures included basal, club, and ciliated cells and were often partially surrounded by smooth muscle actin-expressing and other uncharacterized, mesenchymal cells. The functional significance of the mesenchyme in these cultures has not yet been determined. Nevertheless, the proximity of epithelial and mesenchymal cells in these organoids may provide an in vitro platform to study the complex epithelial-mesenchymal cell interactions in the developing embryonic lung. Alveolar structures in these organoids co-expressed HOPX and SOX9, or SPC and SOX9, consistent with early bipotent alveolar progenitors observed in mice, rather than differentiated alveolar cells [51, 65]. Transcriptome analysis of these human iPSC-derived lung organoids suggested that they are comparable to human foetal lung tissue despite long-term culture of over 3 months [31••]. The future study of epithelial-mesenchymal interactions will likely address this limitation. The same group has recently shown that xenotransplantation of human iPSC-derived lung organoids grown on poly(lactide-co-glycolide) (PLG) scaffolds into the kidney capsule of immunocompromised mice led to enhanced bronchiolar epithelial and mesenchymal organisation compared to organoids grown in vitro. Moreover, in vivo transplanted organoids had improved cellular differentiation of secretory and mesenchymal lineages and associated vasculature [44].

All of these studies illustrate that bronchiolar and alveolar iPSC-derived organoids can be grown in vitro to a degree of maturity which equates to foetal lung tissue. The most successful studies were those which produced organoids consisting of both epithelial and mesenchymal lineages, suggesting that epithelial-mesenchymal cell interaction is crucial for differentiation. Similar results have been found in the differentiation of other organs from human PSCs, including the kidney and intestine [66–68]. Further, maturation and validation by comparison to human adult tissue will be key to determining whether these lung organoids are suitable for future use in disease modelling, modelling of human lung development, and regenerative attempts. Similarly, human iPSC-derived cardiomyocytes display a comparable degree of cellular immaturity (foetal phenotypes), despite 3D culture leading to higher levels of maturity than standard 2D culture. Approaches proposed for improving cardiomyocyte maturity include prolonging the culture period, high oxygen levels, and various combinations of either co-culture or growth factors [69]. Similar approaches could be used for lung organoids. The recent finding that in vivo transplanted lung organoids show improved signs of maturation is highly promising [44]. Although, one hypothesis that remains to be tested is whether the improved epithelial organisation depended on the mesenchyme differentiation or vice versa.

## Organoids Derived from the Adult Lungs

Organoid culture has also been used to investigate stem cell identity and dissect cellular and molecular interactions for the adult lung. Single basal cells isolated from the adult mouse trachea have been grown into organoids, known as tracheospheres, in which the basal cells expand and, following a change in medium composition, differentiate [25]. These cultures could be passaged at least twice, demonstrating the basal cell’s self-renewing ability, and formed part of the evidence that basal cells are adult stem cells. They have subsequently been used successfully to interrogate the roles of Notch, BMP, and IL6 signalling and specific transcription factors in adult basal cells [26, 70, 71•, 72]. Moreover, an elegant in vivo lineage-tracing strategy, combined with tracheosphere derivation, has been used to investigate basal-club cell interactions and demonstrate that in vitro basal cells are able to inhibit the de-differentiation of club cells [73]. The initial derivation of tracheospheres used media compositions that were optimised for expansion and differentiation of basal cells in 2D conditions at air-liquid interface [74]. These are still not completely defined and typically contain serum and bovine pituitary extract. More recently, attempts have been made to improve the growth conditions for basal cells in 2D which would subsequently facilitate organoid experiments. One approach has been to co-culture basal cells with fibroblast cells lines previously used for expansion of epidermis [75]. Another has been to inhibit SMAD signalling which apparently allows the expansion of basal cells from multiple organs, including the mouse and human trachea [76]. These publications raise interesting questions about the signalling interactions of airway basal cells in their in vivo niche which could be partly answered using organoids for co-culture experiments.

The smaller mouse airways consist of secretory club and ciliated cells. Attempts to grow club cells as spheroids rely exclusively on co-culture with underlying stromal populations. For example, isolated club cells could not be grown as spheres alone, only when co-cultured with an ill-defined fibroblast population [77]. Subsequent studies have used similar co-culture techniques and also highlighted the role of FGFR signalling, likely between the mesenchymal and epithelial cells in these co-cultures, demonstrating the utility of such organoid-based co-culture systems for analysing cell-cell signalling in vitro [45]. However, in all cases, the specific identity of the mesenchymal component of these co-cultures is lacking, making systematic analysis of the crosstalk difficult to achieve. By contrast, it has been definitively shown in vitro that co-culture as organoids with lung endothelial cells is sufficient to support the growth and differentiation of the putative BASC stem cells, epithelial cells located in the terminal bronchioles that co-express markers of bronchiolar and alveolar lineages [27••]. This co-culture system has been used to dissect a BMP4-based signalling interaction between the epithelial and endothelial cell types which is sufficient to promote alveolar linage differentiation. Multiple in vivo experiments suggest an intimate relationship between alveolar epithelial, endothelial, and haematopoietic cells, particularly for alveolar repair, which could be dissected in organoid-based systems [78–80].

Multiple in vivo and in vitro experiments have shown that, at steady state, the AC2 cells are the primary stem cell for the alveolar epithelium [28, 51]. Alveolar epithelial organoids, “alveolospheres”, have been generated by co-culturing isolated AC2 cells with a PDGFRA^+^ fibroblast population, which possibly corresponds to the currently ill-defined alveolar lipofibroblasts [28]. This organoid co-culture system has contributed to work showing that AC2 cells are more sensitive to decreases in telomere length than their supporting stromal population and that epithelial stem cell failure is the primary defect in lung diseases caused by telomere syndromes [81]. Further analysis of alveolar organoid co-culture systems will allow the signalling requirements for alveolar epithelial cell growth and differentiation to be determined.

An additional, less well-characterised, distal lung stem cell population contributes to mouse alveolar repair following severe injury, for example that caused by influenza infection. The steady-state characteristics of these cells are not thoroughly defined. However, they have been expanded in 2D, there is consensus that they upregulate the basal cell markers keratin5 (KRT5) and p63 following injury, they are likely to be Sox2-positive, and there is some evidence for multilineage differentiation in organoid culture in vitro [82–85]. A recent publication has characterised a similar, probably identical or over-lapping, distal stem cell population as highly infected by influenza virus, capable of upregulating KRT5 and p63, and highly proliferative in spheroid culture when media was supplemented with FGF ligands, or in the presence of, currently uncharacterised, mesenchymal cells [46]. This is the first description of a spheroid co-culture system for the distal lung stem cell population and, although it has not yet been demonstrated to support self-renewal or differentiation, it is likely to prove highly useful for future mechanistic studies.

For disease modelling, generating mature disease-specific human lung cells is particularly important because murine models often do not completely phenocopy human lung disease. Methods for culturing human airway basal stem cells at air-liquid interface to recapitulate airway epithelial organisation are well-established [86] and have been adapted to include supporting cells, for example fibroblasts and vascular endothelium [87, 88]. Human airway basal cells are also highly amenable to growth and differentiation as organoids [25]. They can be efficiently genetically manipulated in organoid culture [71•], although attempts to further optimise these techniques remain ongoing in multiple labs. Culture of human AC2 cells as organoids has so far been less successful than that for mouse [28] and it is not yet possible to self-renew and differentiate human AC2 cells in 3D cultures.

## Conclusions: The Future of Lung Organoid Research

Organoids have already proved extremely useful for lung research particularly for the study of adult mouse basal cells. In this case, they have provided an initial screening tool prior to pathway functional validation in vivo [26, 70, 72] and also for working out mechanistic details in a simplified model system [73]. It is likely that the transition between using in vitro organoids and in vivo mouse work for different aspects of a project will increase in the future. The new ability to genetically modify organoids in vitro using modern gene-editing techniques, such as CRISPR-Cas9, will enable the functional analysis of genes involved in stem/progenitor self-renewal, differentiation, tissue morphogenesis, and even disease phenotypes [71•]. The rapid and ongoing application of organoid technologies to adult lung stem cells and lung development will undoubtedly facilitate human disease modelling and the more rapid cellular and molecular analysis of genetic variants that have so far only been identified as LOD scores in GWAS studies. Moreover, the development of using simple assays in intestinal organoids to study the response of individual cystic fibrosis patients to specific drugs [89] opens up the possibility of the development of similar assays for some of the highly heterogenous lung diseases, such as idiopathic pulmonary fibrosis. These exciting possibilities are leading to a renaissance in the study of human development and human disease.

One question which still needs to be answered is how complex do we need to make lung organoids for them to be useful? Throughout this review, we have highlighted the use of organoid technology for studying epithelial-epithelial and mesenchymal-epithelial cell interaction. But, should we be using these experiments to develop defined growth media which supports epithelial-only organoids? Or, should we be focusing on the inclusion of multiple cell types in a quest to more completely model the in vivo situation? The answers will most likely depend on the specific research question. However, we are particularly intrigued by the possibilities of using organoid technology to study the relationship between the epithelium and innate immune system or even to model the response to infection. Indeed, a recent report has used human intestinal organoids both as the first in vitro culture system for human norovirus and to study the epithelial response to infection [90]. A similar approach to study *Pseudomonas* infections in lung organoids that model aspects of cystic fibrosis, or bronchiectasis, may be highly fruitful.

The most significant limitations of current research using lung organoids are the lack of reliable validation by comparison to the in vivo cell types and the lack of defined starting populations. Particularly for human, differentiated organoids have been validated by marker expression from the mouse lung literature. An improved understanding of the numerous cell types in the developing and adult human lung is required to improve the validation and maturation attempts for lung organoids and will likely require more single-cell transcriptomics [65, 91]. In particular, a more reliable survey of the various mesenchymal lung cell types will facilitate the use of organoids for studying cell-cell interactions.
